# Supplementary material for: Relative miRNA and mRNA expression involved in arsenic methylation
Source: PLoS One. 2018 Dec 13;13(12):e0209014. doi: 10.1371/journal.pone.0209014 (PMC6292570; doi:10.1371/journal.pone.0209014)
Supplement: S1 Fig — (DOCX) [file pone.0209014.s001.docx]

**Number** ID__________

**Registration form for workers' health**

The first part: the basic situation

1.1 Full name ________ Home address _______________________

Call _________ (Work) ________ (Home)

1.2Sex □ ①Man ②Female

1.3Nation □ ①Han ②Other

1.4One's native heath ______ Province ______City（County）

1.5Date of birth □□□□Year□□Month

1.6Degree of Education □ ①Not going to school ②Primary school ③Junior middle school ④High school ⑤Junior College ⑥Bachelor degree or above

1.7 Marital status□ ①Unmarried ②Married ③Separation ④Divorce ⑤Widowhood

**The second part: Living habits.**

**2.1Daily residence**

Fill out nearly three relocation sites for more than 5 years ①Factory and mine area ②Urban district ③Outside suburb or village

Information near residence：①Smelting plant ②Other plants ③Others___

（1）From _______year____ month to year month **Residence**□ ，Nearby□

（2）From _______year____ month to year month **Residence**□ ，Nearby□

（3）From _______year____ month to year month **Residence**□ ，Nearby□

**2.2Smoking situation**

2.2.1 Your current smoking status is □ ①Smoking ②Used to smoke ③Never （T2.2.3）

2.2.2 If you have quit smoking for more than one year or change in smoking volume, please fill in the paragraph

Start smoking time Termination time Number of years Smoking volume（/day）

（1） ___year_____month to_____year_____month □□ □□

（2）___year_____month to_____year_____month □□ □□

（3）___year_____month to_____year_____month □□ □□

2.2.3 s anyone smoking or working with you? □ ①yes ②no

2.2.4 What you exposed to passive smokers （The smog exhaled smoker for more than 15 minutes / day）is □

①Almost every day□ ②The average is over 3 days / week ③The average is 1to 3 days / week ④The average is lower 1 days / week ⑤No

**2.3Drinking situation**

2.3.1 Your current drinking status is（≧1times/week）□ ①yes ②no ③used to be

2.3.2 How often do you drink? □ ①Everyday or almost everyday ②3-4times/week

③1-2times/week

2.3.3 You choose to drink on most occasion □

①Liquor /one time ②red wine __ Bottle/one time ③Beer ___ Bottle/one time

**2.4Dietary nutrition**

2.4.1Is there a hood or exhaust fan installed? □ ①yes ②no Installation time year month

2.4.2How many times do you cook yourself? □

①Everyday or almost everyday ②3-4 times/week ③1-2 times/week ④average<1time/ween ⑤no

2.4.3 You feel about smoke □ ①light ②middle ③heavy

**2.4 Physical activity assessment**

2.4.1 Do you exercise? □ ①no ②yes

2.4.2 Your average number of exercises per week is □□

2.4.3 The way you exercise most often is □ ①Walk ②Run ③Swimming ④Ball game

⑤Qigong ⑥Others _____

2.4.4 The average time for each exercise is □□Minutes？

**3.1The third part: Family history of disease**

Condition of disease

of family members Father mother Brothers and sisters Other relatives

1. yes，②no） relationship Number

Hypertension ___ ___ ___ ______ ___

coronary heart disease ___ ___ ___ ______ ___

Chronic obstructive

pulmonary disease ___ ___ ___ _______ ___

epilepsy ___ ___ ___ _______ ___

Mental illness ___ ___ ___ _______ ___

Tumour ___ ___ ___ _______ ___

Other diseases ___ ___ ___ _______ ___

**3.2** **Personal disease history**

Name Are you suffering? Initial diagnosis time Current situation

yes no recovery become better Stable Aggravate

Tuberculosis ① ②________year_____month ① ② ③ ④

Chronic

bronchitis ① ②________year_____month ① ② ③ ④

Asthma ① ②________year_____month ① ② ③ ④

Emphysema ① ②________year_____month ① ② ③ ④

Pneumonia ① ②________year_____month ① ② ③ ④

Pneumoconiosis① ②________year_____month ① ② ③ ④

Hypertension ① ②________year_____month ① ② ③ ④

Coronary heart

disease ① ②________year_____month ① ② ③ ④

Cerebral vascular

disease ① ②________year_____month ① ② ③ ④

Dermatitis ① ②________year_____month ① ② ③ ④

Gastritis ① ②________year_____month ① ② ③ ④

Gastric ulcer ① ②________year_____month ① ② ③ ④

Hepatitis ① ②________year_____month ① ② ③ ④

Urinary system

disease ① ②________year_____month ① ② ③ ④

Tumor

(Annotated___)① ②________year_____month ① ② ③ ④

**The fourth part: the history of personal work**

4.1Starting time of work in this plant □□□□ year□□ month

4.2 Termination time of work in this plant □□□□ 年□□ 月

4.3 Current state □ ①On the job ②Transfer ③Retire ④others

4.4 Work history outside the plant □ ① no data ②no ③yes

**The fifth part: the contact history of toxicants and drugs**

**toxicants or**

**drugs** Starting year and month Terminate time Contact situation

Lead □□□□year□□month □□□□year□□month

Carbon

monoxide □□□□year□□month □□□□year□□month

silicon

dioxide □□□□year□□month □□□□year□□month

Radioactive

substance □□□□year□□month □□□□year□□month

Other □□□□year□□month □□□□year□□month

Name of investigator _____________

________year _____ month_____day
