# Supplementary material for: Relative miRNA and mRNA expression involved in arsenic methylation
Source: PLoS One. 2018 Dec 13;13(12):e0209014. doi: 10.1371/journal.pone.0209014 (PMC6292570; doi:10.1371/journal.pone.0209014)
Supplement: S2 Fig — (DOCX) [file pone.0209014.s002.docx]

**编号** ID__________

**工 人 健 康 状 况 登 记 表**

**第一部分 基本情况**

1.1 姓名________ 家庭地址 _______________________

电话 _________ (单位) ________ (家)

1.2性别 □ ①男 ②女

1.3民族 □ ①汉 ②其它

1.4出生地 ______ 省______市（县）

1.5出生日期 □□□□年□□月

1.6文化程度 □ ①未上学 ② 小学 ③初中 ④高中 ⑤大专 ⑥本科及以上

1.7婚姻状态 □ ①未婚 ②已婚 ③分居 ④离婚 ⑤丧偶

**第二部分 居住生活习惯**

**2.1日常居住地**

填写居住5年以上的近三次搬迁地 ①厂矿区 ② 市区 ③矿区外郊区或乡村

居住地附近信息：①冶炼厂 ②其他厂 ③其他_____

（1）从 __________年_____ 月至 年 月 居住在□ ，附近有□

（2）从__________ 年_____月至____ 年___月 居住在□， 附近有□

（3）从 __________年_____月至____ 年___月 居住在□， 附近有□

**2.2吸烟情况**

2.2.1 您目前的吸烟状况是 □ ①目前吸烟 ②以前吸烟 ③从不吸烟 （转2.2.3）

2.2.2 您如有戒烟一年以上或吸烟量变化请分段填写

开始吸烟时间 终止时间 年数 吸烟量（支/天）

（1） _________年_____月 至_________年_____月 □□ □□

（2） _________年_____月 至_________年_____月 □□ □□

（3） _________年_____月 至_________年_____月 □□ □□

2.2.3 和您一起生活或者工作的人中有人吸烟吗？ □ ①是 ②否

2.2.4 您暴露被动吸烟者（接触吸烟者呼出的烟雾超过15分钟/天）情况是 □

①几乎每天□ ②平均≧3天/周 ③平均1-3天/周 ④平均<3天/周 ⑤否

**2.3饮酒情况**

2.3.1您现在（≧1次/周）饮酒情况是□ ①是 ②不是 ③以前曾喝

2.3.2您饮酒频率是 □ ①每天或几乎每天 ②3-4次/周 ③1-2次/周

2.3.3您在大多数场合选择喝 □

①白酒 两/次 ②红酒 _____瓶/次 ③啤酒 _____瓶/次

**2.4饮食营养情况**

2.4.1是否安装了抽油烟机或排气扇 □ ①是 ②否 安装时间 年 月

2.4.2您自己做饭次数 □

①每天或几乎每天 ②3-4次/周 ③1-2 次/周 ④平均<1次每周 ⑤否

2.4.3你感觉油烟 □ ①轻 ②中 ③重

**2.5 体力活动评估**

2.4.1您进行锻炼吗？ □ ①否 ②是

2.4.2您平均每周锻炼次数为 □□

2.4.3您最常锻炼方式是 □ ①走路 ②跑步 ③游泳 ④球类运动 ⑤气功 ⑥其他 _____

2.4.4 平均每次锻炼的时间是 □□分钟？

**第三部分 疾病史**

**3.1家族疾病史**

家族成员患病情况 父亲 母亲 兄弟姐妹 其他亲属

（①是，②否） 关系 人数

高血压 ___ ___ ___ _______ ___

冠心病 ___ ___ ___ _______ ___

慢性阻塞性肺病 ___ ___ ___ _______ ___

癫痫 ___ ___ ___ _______ ___

精神性疾病 ___ ___ ___ _______ ___

肿瘤 ___ ___ ___ _______ ___

其他疾病 _______ _______ _______ _______

**3.2个人疾病史**

名称 是否患以下疾病 初次诊断时间 目前状况

是 否 痊愈 好转 稳定 加重

结核 ① ② _________年_____月 ① ② ③ ④

慢性支气管炎 ① ② _________年_____月 ① ② ③ ④

哮喘 ① ② _________年_____月 ① ② ③ ④

肺气肿 ① ② _________年_____月 ① ② ③ ④

肺炎 ① ② _________年_____月 ① ② ③ ④

尘肺 ① ② _________年_____月 ① ② ③ ④

高血压 ① ② _________年_____月 ① ② ③ ④

冠心病 ① ② _________年__ __月 ① ② ③ ④

脑血管病 ① ② _________年_____月 ① ② ③ ④

皮炎 ① ② _________年_____月 ① ② ③ ④

胃炎 ① ② _________年_____月 ① ② ③ ④

胃溃疡 ① ② _________年_____月 ① ② ③ ④

肝炎 ① ② _________年_____月 ① ② ③ ④

泌尿系病 ① ② _________年_____月 ① ② ③ ④

肿瘤(注明____)① ② _________年_____月 ① ② ③ ④

**第四部分 个人工作史**

4.1本厂矿开始工作时间 □□□□ 年□□ 月

4.2本厂矿终止工作时间 □□□□ 年□□ 月

4.3 目前状态 □ ①在职 ②调离 ③退休 ④其他

4.4 本厂矿外工作史 □ ① 缺资料 ②无 ③有

**第五部分 毒物和药物接触史**

毒物或药物 开始年月 终止年月 接触情况

铅 □□□□年□□月 □□□□年□□月

一氧化碳 □□□□年□□月 □□□□年□□月

二氧化硅 □□□□年□□月 □□□□年□□月

放射性物质 □□□□年□□月 □□□□年□□月

其他 □□□□年□□月 □□□□年□□月

调查者姓名 _____________

________ 年 _____ 月_____日
