# Supplementary material for: Relative miRNA and mRNA expression involved in arsenic methylation
Source: PLoS One. 2018 Dec 13;13(12):e0209014. doi: 10.1371/journal.pone.0209014 (PMC6292570; doi:10.1371/journal.pone.0209014)
Supplement: S1 File — (DOC) [file pone.0209014.s003.doc]

**Relative miRNA and mRNA Expression Involved in Arsenic Methylation**

Huirong Cheng^1^, Pei Hu^1^,Weihua Wen^1^**, Ling Liu^2^*

^1^Department of Occupational Health, Yunnan Provincial Center for Disease Control and Prevention. No.158, Dongsi Street, Kunming, Yunnan, 650022, China;

^2^ First affiliated hospital of Kunming medical university, Kunming 650032, People’s Republic of China.

* Corresponding to L. Liu, E-mail address:54319937@qq.com. Respiratory Medicine Ward II, First affiliated hospital of Kunming medical university, Kunming 650032, People’s Republic of China.

**Corresponding to W.H. Wen, E-mail address: Dongsijiehua@sina.com. Yunnan Provincial Center for Disease Control and Prevention. No.158, Dongsi Street, Kunming, Yunnan, 650022, People’s Republic of China.

**Abstract**

Three arsenic species in urine are measured using an atomic absorption spectrophotometer. RT-PCR is performed to detect the expression levels of AS3MT, 3 miRNAs, and 17 relative mRNAs in 43 workers who produce arsenic trioxide, 36 workers who stopped exposure to arsenic for 85 days, and 24 individuals as the control group. The concentrations of urinary arsenic are very high in workers. A negative correlation between AS3MT and miRNA548 is found. There exist significant changes for most relative miRNAs and mRNAs in workers. There are no significant differences between workers who stopped exposure to arsenic and the control group for most selected miRNAs and mRNAs, but the miRNA548 levels show significant changes. Similar positive correlations between the expression of AS3MT and all selected mRNAs are observed, and a negative correlation between the expression of miRNA548 and many relative mRNAs is found as well. AS3MT and miRNA548 may regulate arsenic methylation jointly, which when involved in a group of relative mRNAs may play roles in arsenic metabolism and epigenetic changes caused by this metabolism.

**Key words**: Arsenic; AS3MT; MiR548; MiRNAs

**Introduction**

Arsenic can promote cancer through epigenetic mechanisms, and AS3MT plays a role in this process ^[1]^. AS3MT is regulated by some miRNAs. Many mRNAs are closely involved in miRNA biogenesis pathways and their regulation ^[2]^. Relative genes associated with those miRNAs and relative mRNAs are widely distributed in the genome, and most are closely involved in malignant tumors ^[1, 3-5]^.

Arsenic exposure disrupts the genome-wide expression of miRNAs in vivo, which can lead to changes in gene expression ^[6]^. A list of miRNAs whose expression levels are known to be affected by iAs treatment exists, corroborating the importance of proceeding with the hunt for specific subset of miRNAs that can serve as potential biomarkers of iAs effects and that have useful diagnostic value^[7]^. The MiR-548 family has been demonstrated to be involved in the pathogenesis of several cancers; however, its role has not yet been elucidated ^[8]^. This miRNA family is less expressed in some patients and plays an important role in some cancers ^(9)^.

The miRNA processing pathway has long been viewed as linear and universal to all mammalian miRNAs ^[2]^. Some relative mRNAs, such as exportin-5, dicer, trbp, Argonaute (Ago2), Lin28 (also termed Lin28a), and Lin28b, play roles in this process ^[2,5,10]^. Arsenic exposure has been shown to alter methylation levels of both global DNA and gene promoters, which may cause changes in expression for many relative mRNAs to promote cell apoptosis, cell cycle inhibition, or relative regulation of carcinogenesis ^[11, 12]^. There are many relationships among the above mRNAs.

Although experiments in suitable model systems could complement the human studies, there may be differences in epigenetic effects between animals and humans and between various tissues and cell types ^[13]^. Thus, studies on human populations exposed to high levels of arsenic are necessary to understand individual differences between arsenic methylation and genetic background.

In this study, we investigate the effects of arsenic exposure, defined by the internal biomarkers inorganic arsenic (iAs), monomethylarsonic acid (MMA) and dimethylarsinic acid (DMA) in urine, on the expression of AS3MT, 3 miRNAs and 17 relative mRNAs. We explore how miRNAs and relative mRNAs play a role in AS3MT activity and arsenic metabolism, and the relationships among the expression levels of different RNAs in workers who produce arsenic trioxide, to study the widespread roles of AS3MT and miRNAs networks in workers exposed to arsenic trioxide.

**Materials and Methods**

**Study population**

Our analysis complies with the Declaration of Helsinki Ethical Principles for Medical Research Involving Human Subjects (World Medical Association 1989), and the Declaration of Helsinki (1964, amended in 1975, 1983, 1989, 1996 and 2000) of the World Medical Association. Questionnaires were used to obtain information from each individual including age, sex, type of work, service length, smoking and alcohol use (current smoker who smokes more than 10 cigarettes each day and any consumption of alcohol), other potential chemical exposures (lead, carbon monoxide, silicon dioxide, and so on), and data on health status, dietary habits, history of chronic disease, family member history, place of birth, race, education, economic conditions, and reproductive conditions. This study was approved by the Ethics Committee of the Yunnan Provincial Center for Disease Control and Prevention. Individuals were enrolled in the study after agreeing to participate and signing an Informed Consent form. Completion of the questionnaire was entirely voluntary.

The data used for our analysis were from workers in two plants. We collected the data from 43 workers in one arsenic plant that was producing arsenic trioxide (named A plant), and 36 workers from another plant in which the production had been stopped 85 days from the time biological samples were collected (named B plant). The plants chosen for our study produce arsenic trioxide using arsenic ore by reverberator smelting and charcoal deoxidizing. Based on the characteristics of the chemical components of the ore and the production technique flow adopted, there are few other occupational hazard factors except for arsenic pollution in the plant. The occupational protective measures of the plant are far from being sufficient and efficient. Additionally, the 24 individuals in the control group resided in villages that were located more than 50 km in distance from each of the two plants and had similar social economic status to the 79 workers. The prefecture of the city to which the two plants belong is well known for having a high incidence of lung cancer, and arsenic may be involved in the process.

**Sample collection**

Written instructions regarding the hygienic conditions for collection of samples and 500 ml polyethylene containers treated with hydrochloric acid and rinsed with deionized water were provided to all participants. Subjects were asked to provide the first morning void urine. At the same time, blood samples were collected, and total RNA (1 mg) was extracted within 1 day.

**Determination of As metabolisms**

We determined As species (iAs, MMA, and DMA) in urine using an atomic absorption spectrophotometer (AA-6800) with an As speciation pretreatment system (ASA-2SP, Shimadzu Co., Kyoto, Japan). Speciation analysis was based on the well-established hydride generation of volatile arsines, followed by cryogenic separation in liquid nitrogen. The limit of detection of 1 ng±<5% for each of the three As species was determined using hydride generation-atomic absorption spectrometry (HGAAS). Briefly, 1 ml urine that had been stored at -80°C was thawed at room temperature and digested with 2 N NaOH at 100°C for 3 hrs in a 15-ml polymethylpentene test tube (Sarstedt), followed by dilution with Milli-Q water (Millipore, Yonezawa, Japan). This digestion procedure did not alter the distribution of iAs or methylated arsenicals (Yamauchi and Yamamura. 1984). The absorbance of As in the digested urine samples was determined at 193.7 nm.

**Quantitative real-time PCR analysis**

Overall, 21 RNAs including AS3MT, 3 miRNAs, 6 relative mRNAs that are important in the miRNA biogenesis pathways and their regulation, and 11 relative mRNAs that promote cell apoptosis, cell cycle inhibition, or relative regulation of carcinogenesis, as well as a *β*-actin sequence (control fragment), were selected in this study. Total RNA (1 mg) was extracted using Trizol reagent (Invitrogen) following manufacturer’s instruction, and then transcribed into cDNA with NCode™ VILO™ miRNA cDNA Synthesis Kit (Invitrogen). Quantitative real-time PCR (qRT-PCR) was performed with the Platinum® SYBR® Green qPCR SuperMix-UDG (Invitrogen) in ABI7900 (Applied Biosystems, America). PCR primers are designed for all RNAs. The relative expression levels of all RNAs were determined using the 2*^-△△Ct^* method. All reactions were performed in triplicate.

**Statistical analysis**

All statistical analyses were performed using SPSS software (Version 19, Chicago, IL, USA). The concentrations of iAs, MMA and DMA were first log transformed to improve the normality of measures but then transformed back to the arithmetic scale for reporting purposes. After assessing the association among the levels of miRNAs, relative mRNAs and three arsenic species by Spearman’s rank correlation analysis, covariance and independent samples *t*-test were performed for the analysis of arsenic species and all RNAs among groups under different levels of arsenic trioxide exposure. We then investigated the association of arsenic species and all RNAs among groups under different levels of arsenic trioxide exposure using multivariate linear regression models with adjustment for age, sex, smoking status, work years, and urinary creatinine. All statistical tests were two-sided, with a *P* value< 0.05 considered statistically significant for any single analysis.

**Results**

There are no significant differences in sex, smoking, alcohol consumption, and so on among the exposed workers in the two plants and the control group. For the demographics of the study population, see Table 1.

**Changes in iAs species and many RNAs**

The concentrations of iAs, MMA and DMA in urine are shown in Figure 1. Compared to the control group, the increases in the levels of iAs, MMA, and DMA in urine of workers exposed to arsenic are all statistically significant (*p*<0.01).

The expression levels of 21 RNAs are shown in Figure 2. Compared to that in the control group, *AS3MT* expression shows significant changes for workers producing arsenic (*P*<0.01), but not for workers who stopped exposure to arsenic for 85 days. Based on genome informatics analysis, it is MiR-5*48c-3p* that may regulate *AS3MT* expression. Compared with that in the control group, the decreases in MiR-548-3p expression in workers from the two plants are statistically significant (*P*<0.01).

Lin28, Lin28b, Dicer, Expothin-5, Trbp and Ago2 are mRNAs that are involved in the generation and maturation of miRNAs. Compared to those in the control group, the relative mRNA levels increase significantly for workers producing arsenic (*P*<0.05), but not for workers who stopped exposure to arsenic for 85 days.

Compared to those in the control group, there are significant changes for 9 mRNAs that promote cell apoptosis, cell cycle inhibition, or relative regulation of carcinogenesis for workers producing arsenic (*p*<0.01), but only changes in *p*15 and Puma expression for workers who stopped exposure to arsenic for 85 days (*P*<0.05). It is worth noting that the levels of *noxa* expression in workers who stopped exposure to arsenic for 85 days are lower than that of the control group (*p*<0.01).

**Spearman’s rank correlation analysis among arsenic species, relative mRNAs and miRNAs networks**

The concentrations of iAs, MMA and DMA in urine are positively correlated with the expression levels of *AS3MT* RNA (*P*<0.05) and negatively correlated with those of MiR-548-3p (*p*<0.01). The expression levels of *AS3MT* RNA are negatively correlated with those of MiR-548-3p (*p*<0.05). The expression levels of *AS3MT* RNA are positively correlated with those of all relative mRNAs (*p*<0.05), while the levels of MiR-548-3p are negatively correlated with those of many relative mRNAs (*p*<0.05). With the increase in the content of iAs, MMA and DMA in urine, an increase in the expression levels for many relative mRNAs is observed (*p*<0.05). See Table 2.

**Discussion**

It is of great relevance that biomethylation plays a role in activating cancer and inducing toxicity for inorganic arsenic. The methylation of iAs yields methylated metabolites in which arsenic is present in both pentavalent and trivalent forms ^[14]^. Studies show that arsenic exposure and the incomplete methylation capacity of arsenic are adversely associated with cancer and other diseases ^[15-17]^. AS3MT is the key enzyme in the biotransformation pathway, catalyzing the methylation of inorganic arsenic and playing an important role in the metabolism of this metalloid ^[2,3,18]^.

Our previous study showed that arsenic levels at 7 posts in the plant producing arsenic trioxide were very high and that the workers had been exposed to high arsenic levels for long periods of time ^[15]^. Relative concentrations and proportions of arsenic metabolites in urine have been identified as potential biomarkers of susceptibility to iAs toxicity [16,19].

Acute arsenic exposure at the levels observed in poisoned Japanese patients has been associated with a high concentration of 8-OHdG, which may result from DNA damage caused by arsenic metabolism ^[20]^. The data indicate that some aspects of chronic and acute arsenic poisoning may be reversible with the cessation of exposure. Consistent with Yamauchi et al., our results show that there are substantial changes in the expression of many relative mRNAs that are important in the miRNA biogenesis pathways and their regulation and that promote cell apoptosis, cell cycle inhibition, or relative regulation of carcinogenesis. These RNAs may play a role in promoting cancer and inducing toxicity, which are probably involved in arsenic metabolism similar to AS3MT.

There exist aberrant changes for many relative mRNAs from workers in the two plants, and these changes cannot not be explained using differences in arsenic concentration only. *AS3MT* and MiR-548-3p may play roles in arsenic metabolism, promoting cancer and inducing toxicity by multiple pathways.

MiRNAs are involved in temporal and tissue-specific eukaryotic gene regulation, either by translational inhibition or exonucleolytic mRNA decay ^[21]^. Altered miRNA expression may lead to widespread gene expression changes ^[22]^. Although the mode of action of miRNAs has attracted great attention, the principles governing their expression and activity are only beginning to emerge. There are many branches, crossroads and detours in miRNA processing, according to recent studies _[1]_. In this study, the expression levels of selected relative mRNAs are likely under the control of transcription factors, for example, p53 ^[23, 24]^. Relative mRNAs, such as Lin28 and Dicer, which are important in the generation and maturation of miRNAs, may be involved in the metabolism of arsenic by ASMT, miR-548-3p, *p*53, and so on.

Based on genome informatics analysis, it is miR-548c-3p that very likely regulates AS3MT. Our preinvestigation shows that there are obvious changes in the expression levels of miR-548-3p in workers exposed to arsenic, and we plan to explore the expression rules of this miRNA and the role it plays in cancers associated in arsenic exposure. The current results show that there is lower expression of mir548 and higher expression of AS3MT in workers exposed to arsenic. The miRNA processing pathway may be closely involved in this process.

Both AS3MT and miR-548-3p may be highly involved in metabolizing arsenic, activating cancer and inducing toxicity, but they may play their roles in different ways. Most likely, AS3MT has close relations with many relative mRNAs, including all mRNAs selected in this study. At the same time, MiR-548-3p is closely related to some mRNAs that may be closely involved in AS3MT functions, such as Lin28, Exportin-5 and Ago2. AS3MT function may be influenced by not only MiR-548-3p but also many other RNAs. The biogenesis pathways of miR-548c-3p and other miRNAs, as well as their generation and maturation, are involved in many pathways that may play an important role in arsenic metabolism and epigenetic changes.

**Conclusions**

Our analysis in this study suggests potentially widespread changes in the expression levels of *AS3MT*, miRNAs and relative mRNAs in workers, which may be caused by arsenic metabolism. *AS3MT* and MiR-548-3p likely have important roles in arsenic methylation and epigenetic changes, but in different ways, and a group of relative mRNAs also take part in this process.

**COMPETING FINANCIAL INTERESTS**

The authors declare that they have no competing interests.

**FUNDING**

This study was supported by National Natural Science Foundation of China (NSFC) research grant (30860238 and 81160343), Applied Basic Research Planning of Yunnan Province (2011FB147), China Scholarship Council, and Personnel Training Special Funds on High Level Health Technology of Yunnan Province (D-201250).

**Acknowledgments:** We thank all individuals who volunteered to participate in this study.

**REFERENCES**

1 Engström K, Vahter M, Mlakar SJ, Concha G, Nermell B, Raqib R, et al ( 2011) Polymorphisms in Arsenic(+III oxidationState) Methyltransferase (AS3MT) Predict Gene Expression of AS3MT as Well asArsenic Metabolism. Environ Health Persp. 119: 182-188.

2 Winter J, Jung S, Keller S, Gregory RI, Diederichs S (2009)Many roads to maturity: microRNA biogenesis pathways and their regulation. Nat. cell biol. 11:228-234.

3 Fujihara J, Soejima M, Yasuda T, Koda Y, Agusa T, Kunito T, et al(2010)Global analysis of genetic variation in human arsenic (+3 oxidation state) methyltransferase (AS3MT). Toxicol Appl Pharm. 243:292-299.

4 Chitkara D, Mittal A, Mahato RI (2015)miRNAs in pancreatic cancer: Therapeutic potential, delivery challenges and strategies, Adv Drug Deliv Rev. 81:75-93.

5 Valenzuela-Miranda D, Nuñez-Acuña G, Valenzuela-MuñozV, Asgari S, Gallardo-Escárate C(2015)MicroRNA biogenesis pathway from the salmon louse (Caligus rogercresseyi): Emerging role in delousing drug response, gene, 555(2):231-241.

6 Ren X, Gaile DP, Gong Z, Qiu W, Ge Y, Zhang C, et al(2015) Arsenic responsive microRNAs in vivo and their potential involvement in arsenic-induced oxidative stress Toxicol Appl Pharmacol. 283(3):198-209. .

7 Sturchio E, Colombo T, Boccia P, Carucci N, Meconi C, Minoia C, Macino G. (2013) Arsenic exposure triggers a shift in microRNA expression.Sci Total Environ.472:672-80.

8 Shi YF, QiuM, WuYY, HaiL. (2015) MiR-548-3p functions as an anti-oncogenic regulator in breast cancer. Biomedicine & Pharmacotherapy, 75:111-116.

9 Tahiri A,LeivonenSK, Lüders T,SteinfeldI, AureMR, GeislerJ, et al(2014) Deregulation of cancer-related miRNAs is a common event in both benign and malignant human breast tumors. Carcinogenesis 35 (1):76-85.

10Zhou JB, Ng SB, Chng WJ (2013)LIN28/LIN28B: An emerging oncogenic driver in cancer stem cells,Inter. J. Biochem. Cell B. 45(5):973-978.

11 Yang TY, Hsu LI, Chiu AW, Pu YC, Wang SH, Liao YT, et al (2014) Comparison of genome-wide DNA methylation in urothelial carcinomas of patients with and without arsenic exposure, Environ. Res. 12857-63.

12 Reichard JF, enburger MS, Puga A (2007)Long term low-dose arsenic exposure induces loss of DNA methylation. Biochem Biophys Res Commun. 352: 188–192.

13 KilleP, AndreJ, AndersonC , AngHN, BrufordMW,Bundy JG, et al (2013) DNA sequence variation and methylation in an arsenic tolerant earthworm population. Soil. Biol. Biochem. 57:524-532.

14 Styblo M, Del Razo LM Vega L, Germolec DR, LeCluyse EL, Hamilton GA, Reed W, et al(2000) Comparative toxicity of trivalent and pentavalent inorganic and methylated arsenicals in rat and human cells.Arch Toxicol. 74(6):289-99.

15 Yu RC, Hsu KH, Chen CJ, Froines JR.（2000）Arsenic methylation capacity and skin cancer.Cancer Epidemiol Biomarkers Prev. 9(11):1259-62.16 Wen JH, Wen WH, Li L, Liu H (2012)Methylation capacity of arsenic and skin lesions in smelterplant workers, Environ. Appl.Pharmacol.34:624-630.

17 Wen WH, Wen JH, Lu L, Liu H, Yang J, Cheng HR, et al(2011) Metabolites of arsenic

and increased DNA damage of p53 gene in arsenicplant workers, Toxicol. Appl. Pharmacol. 254, 41-47.

18 Song XL, Geng ZR, Li XL, ZhaoQ, Hu X, Zhang XR, et al(2011)Functional and structural evaluation of cysteine residues in the human arsenic (3 oxidation state) methyltransferase (hAS3MT). Biochimie93:369-375.

19AntonelliR, Shao K, Thomas DJ, II BS, Cowden J (2014)AS3MT, GSTO, and PNP polymorphisms: Impact on arsenic methylation and implications for disease susceptibility.Environ. Res. 132:156-167.

20Yamauchi H, Aminaka Y, Yoshida K, Sun GF, Pi JB, Waalkes MP(2004)Evaluation of DNA damage in patients with arsenic poisoning: urinary 8-hydroxydeoxyguanine. Toxicol. Appl. Pharmacol. 198, 291-296.

21Iorio MV, Casalini P, Piovan C, Braccioli L, Tagliabue E (2011)Breast cancer and microRNAs: therapeutic impact. The Breast. 20:63-70.

22Sonkoly E, Pivarcsi E (2011)MicroRNAs in inflammation and response to injuries induced by environmental pollution. Mut Res.717:46-53.

23O’Donnell KA, Wentzel E A, Zeller KI, Dang CV, Mendell JT (2005)c-Myc-regulated microRNAs modulateE2F1 expression. Nature435, 839–843.

24He L, He XY, Lim LP, Stanchina ED, Xuan ZY (2007) A microRNA component of the p53 tumour suppressor network. Nature447:1130–1134.
